# Molecular and clinical aspects relevant for counseling individuals with clonal hematopoiesis of indeterminate potential

**DOI:** 10.3389/fonc.2023.1303785

**Published:** 2023-12-15

**Authors:** Anna Maria Cacic, Felicitas Isabel Schulz, Ulrich Germing, Sascha Dietrich, Norbert Gattermann

**Affiliations:** ^1^ Department of Hematology, Oncology and Clinical Immunology, Medical Faculty and University Hospital Düsseldorf, Heinrich Heine Universität Düsseldorf, Düsseldorf, Germany; ^2^ Center for Integrated Oncology Aachen Bonn Cologne Düsseldorf (CIO ABCD), Düsseldorf, Germany

**Keywords:** clonal hematopoiesis of indeterminate potential (CHIP), preleukemia, myelodysplastic syndromes, germline mutations, PARP inhibitors, mutation analysis, cardiovascular risk factors, genetic counseling

## Abstract

Clonal hematopoiesis of indeterminate potential (CHIP) has fascinated the medical community for some time. Discovered about a decade ago, this phenomenon links age-related alterations in hematopoiesis not only to the later development of hematological malignancies but also to an increased risk of early-onset cardiovascular disease and some other disorders. CHIP is detected in the blood and is characterized by clonally expanded somatic mutations in cancer-associated genes, predisposing to the development of hematologic neoplasms such as MDS and AML. CHIP-associated mutations often involve DNA damage repair genes and are frequently observed following prior cytotoxic cancer therapy. Genetic predisposition seems to be a contributing factor. It came as a surprise that CHIP significantly elevates the risk of myocardial infarction and stroke, and also contributes to heart failure and pulmonary hypertension. Meanwhile, evidence of mutant clonal macrophages in vessel walls and organ parenchyma helps to explain the pathophysiology. Besides aging, there are some risk factors promoting the appearance of CHIP, such as smoking, chronic inflammation, chronic sleep deprivation, and high birth weight. This article describes fundamental aspects of CHIP and explains its association with hematologic malignancies, cardiovascular disorders, and other medical conditions, while also exploring potential progress in the clinical management of affected individuals. While it is important to diagnose conditions that can lead to adverse, but potentially preventable, effects, it is equally important not to stress patients by confronting them with disconcerting findings that cannot be remedied. Individuals with diagnosed or suspected CHIP should receive counseling in a specialized outpatient clinic, where professionals from relevant medical specialties may help them to avoid the development of CHIP-related health problems. Unfortunately, useful treatments and clinical guidelines for managing CHIP are still largely lacking. However, there are some promising approaches regarding the management of cardiovascular disease risk. In the future, strategies aimed at restoration of gene function or inhibition of inflammatory mediators may become an option.

## Introduction

Clonal hematopoiesis (CH) is characterized by the presence of a population of blood cells derived from a mutated multipotent stem/progenitor cell that has gained an abnormal growth advantage.

Specifically, clonal hematopoiesis of indeterminate potential (CHIP), as defined by the new WHO classification of hematolymphoid tumors, refers to CH with somatic mutations in myeloid malignancy-associated genes detected in the blood or bone marrow at a variant allele frequency (VAF) of ≥2% in individuals without diagnosed hematologic disorder or unexplained cytopenia (1–3). It should be emphasized that CHIP is not yet a disease, since the accepted definition excludes persistent (≥4 months) cytopenia and an overt pathology associated with the somatic lesion (3, 4). Using a VAF of ≥2% as cut-off, up to 20% of individuals above the age of 70 have detectable CH (5–7). Considering the demography in Germany, it is estimated that 2.75 million people are affected (8). Regarding the individual person with CH, intraindividual heterogeneity has been reported, with disparities observed between affected cell lineages, tissue compartments (peripheral blood *vs*. bone marrow), and anatomic locations (9). CHIP mutations can arise in both the myeloid lineage (referred to as M-CHIP) and the lymphoid lineage (known as L-CHIP) of the hematopoietic system (10). Both myeloid and lymphoid somatic gene mutations, as well as myeloid and lymphoid mosaic chromosomal alterations (mCAs), are associated with the risk of lineage-specific hematologic malignancies (10). In the case of M-CHIP, the majority of mutations are found in three genes, namely *DNMT3A*, *TET2*, and *ASXL1 (* 10). Somatic mutations in myeloid-biased CH are correlated with an elevated risk of myeloid malignancy, cardiovascular disease (CVD), and all-cause mortality (11). L-CHIP appears to be distributed more evenly across a larger number of genes, resembling the distribution of variants in the remaining M-CHIP genes (10). Somatic mutations in lymphoid-biased CH are associated with the development of lymphoid malignancies and late-onset manifestations of autoimmunity and immune deficiency (11).

The CHIP clones identified so far exhibit a range of large-scale mCAs, including deletions, duplications, and copy-neutral loss of heterozygosity (CN-LOH) on all chromosomes (12–16). mCA-driven CH primarily predisposes to lymphoid malignancies such as chronic lymphocytic leukemia (CLL) and is associated with a twofold increase in all-cause mortality (16, 17). Notably, events involving *JAK2-*mutated CN-LOH have been demonstrated to significantly elevate cardiovascular risk (17). Additionally, mCA-associated CH may lead to impaired immune function, making individuals more susceptible to infections (18).

Again, it is important to note that the term CHIP should only be applied to individuals with normal blood counts (19). In cases where CHIP is identified alongside one or more persistent cytopenias that are otherwise unexplained by hematologic or non-hematologic conditions and that do not meet diagnostic criteria for defined myeloid neoplasms (MNs), the condition is referred to as CCUS (clonal cytopenia of undetermined significance) (1). Both CCUS and CHIP become more prevalent with age and are relatively common among elderly individuals (2). Patients with CHIP have an elevated risk of developing a MN compared to controls without CHIP (3, 5, 6, 20–23), and they also have an increased risk of developing progressive atherosclerosis and related cardiovascular disorders (5, 24). However, it should be noted that in the majority of patients with CHIP, neither malignancy nor severe CVD manifests (19).

Understanding the intricate interplay between genetic predisposition, somatic mutations, and environmental influences is crucial for unraveling the complexities of CHIP and its implications for health. Genetic predisposition seems to play a significant role, contributing to the accumulation of somatic mutations in hematopoietic stem cells (HSCs). Some of these mutations are key drivers of CHIP. Acquisition of CH mutations occurs through natural mechanisms during aging, such as point mutations due to spontaneous deamination of 5-methylcytosine to thymine (25), or less commonly through errors during repair of double-stranded DNA breaks, creating small insertions/deletions (indels), or through replication errors produced by DNA polymerase (26).

CH is associated with inflammation that promotes HSC expansion, increasing the risk of hematologic and cardiologic diseases. This relationship is believed to create a “CH expansion-inflammation cycle” (27, 28). When a stem cell acquires a CH-associated gene mutation, it gains enhanced fitness in an inflammatory microenvironment, leading to CH-mutant hematopoietic stem and progenitor cell (HSPC) expansion. In turn, this results in increased and chronic expression of proinflammatory cytokines and other mediators that alter the hematopoietic milieu, creating an environment that suppresses normal HSPCs and establishes a vicious cycle of enhanced CH cell fitness and inflammation. Targeting inflammatory mechanisms may disrupt this cycle, reducing the risk of cancer and comorbid inflammatory diseases.

## CHIP and hematologic risks

The accumulation of somatic cancer-associated mutations in HSCs, as observed in CHIP, enhances the risk of developing a hematologic neoplasm such as myelodysplastic syndrome (MDS) or acute myeloid leukemia (AML). Presumably, this leukemic transformation is driven by exaggerated stimulation of inflammatory processes in the bone marrow, coupled with inflammation associated with aging, ultimately resulting in the functional impairment of HSCs (29). Usually, the development of myeloid malignancies involves progression through certain precursor stages (CH, CHIP, CCUS).

Early-stage CH is characterized by the initial acquisition of a somatic mutation in a leukemia-associated gene, resulting in improved fitness and clonal expansion of the affected stem cell (30). The most frequent gene mutations associated with CHIP are *DNMT3A*, *TET2*, and *ASXL1* (DTA genes). Individuals affected by these mutations in the context of CHIP exhibit no evidence of hematologic malignancy or cytopenia (31). Such mutations are detectable in approximately 5% of individuals aged 60-69 years, and about 10% of those aged 70-79 years (5, 32). Driver mutations associated with CHIP exhibit distinct patterns of clonal expansion over time. Previous findings indicate that *DNMT3A*-mutant clones are more likely to expand earlier in life (33). While clones with mutations in *DNMT3A* and *TP53* display the slowest annual growth rate at 5%, clonal lineages with mutations in *TET2*, *ASXL1*, *PPM1D* and *SF3B1* expand at approximately twice the rate, and those with mutations in splicing factors such as *SRSF2*, *PTPN11*, and *U2AF1* grow at an average annual rate of 15-20% (33). The rapid growth of the latter set of mutations may contribute to the observed worse prognoses and increased disease severity associated with CHIP mutations in splicing factor genes (34). The risk of hematologic malignancy appears to be determined by the size of the clone as well as the type and number of mutated genes. The presence of mutations in spliceosome genes, *IDH1/2* mutations, *TP53* mutations, multiple mutations and a high VAF are associated with an increased risk of myeloid malignancies, whereas the risk appears to be lowest in patients with single *DNMT3A* or *TET2* mutations (35). Advanced precursor states preceding the development of MDS and AML include CCUS and ICUS. As expected, CCUS, which carries a detectable clonal marker, has a significantly poorer prognosis than ICUS (31), with a considerable risk of transformation to MDS/AML (36). As an alternative to extensive mutation screening based on next-generation DNA sequencing, HUMARA (human androgen receptor gene-based assay) can be harnessed to assess clonality and thus differentiate between CCUS/MDS and ICUS (37).

The risk of malignant transformation is relatively low for CHIP, with a yearly progression rate of 0.5-1.0% (5, 24). ICUS carries a 9% probability of malignant clonal evolution at both 5 years and 10 years, while CCUS shows a probability of 82% at 5 years and 95% at 10 years (36). The outcome in CCUS is influenced by several clone metrics, including the number of mutations and specific genes affected (31). Epigenetic modifier genes *DNMT3A*, *TET2*, and *ASXL1* are commonly mutated in CCUS but confer a lower risk, while spliceosome genes *U2AF1*, *SRSF2*, and *SF3B1* are associated with the highest risk of progression (31, 36). A VAF >10% is associated with a 50-fold increase in the likelihood of progression (5). CCUS with a VAF of more than 20%, combined with mutations in spliceosome genes and DTA mutations, is considered “high-risk CCUS” and carries a 5-year cumulative probability of progression of 95% (36). Further research is needed to elucidate the mechanisms of disease progression and identify predictive markers that can guide prognostication and treatment decisions in individuals with CH, CHIP, CCUS, and ICUS.

An increased prevalence of CHIP has been observed following cytotoxic therapies such as chemotherapy, radiotherapy, and PARP inhibitor therapy (38–41), suggesting that these treatments contribute to the emergence and expansion of mutated cells in the hematopoietic system, thereby fostering the development of therapy-related myeloid neoplasms (t-MNs). In particular, cancer therapy involving radiation, platinum, and topoisomerase II inhibitors shows a preference for selecting mutations in DNA damage response (DDR) genes such as *TP53*, *PPM1D* and *CHEK2 (* 38). Screening for CHIP in individuals who have undergone genotoxic treatment may help to identify those who have an elevated risk of developing a hematologic malignancy.

In addition to single-nucleotide variants (SNVs) and small indels, which contribute to the risk of malignancy in the context of CHIP, large structural changes to chromosomes, namely mCAs, can also play a role in driving clonal expansion (12, 13). Recent research has revealed that the presence of either SNVs/indels alone or mCAs alone is associated with a relatively low risk or cancer transformation (10, 42). However, when both forms of CH exist simultaneously, there is a greater risk of cancer transformation (10, 42). Specific forms of mCAs may predispose individuals to L-CHIP, while other forms are more likely to result in M-CHIP (10).

## CHIP and cardiovascular risks

Myeloid precursor conditions, especially CHIP, have been linked to increased all-cause mortality (5, 6, 16, 32), predominantly attributed to cardiovascular causes, including coronary artery disease and stroke (5, 16, 43). CHIP mutations in genes such as *DNMT3A, TET2, ASXL1, TP53, JAK2*, and *SF3B1* have been identified that are particularly associated with an increased risk of developing cardiovascular diseases (44). **Table 1** presents the major cardiovascular conditions and their corresponding CHIP-associated mutations, according to current literature.

**Table 1:** CHIP and diverse cardiovascular conditions.

Heart disease	Related CHIP-mutations
**Cardiovascular atherosclerotic disease**	*DNMT3A* (45)
	*TET2* (24, 46, 47)
	*JAK2^V617F^* (48–50)
	*SF3B1* (47)
	*SRSF2* (47)
	*U2AF1* (47)
**Early-onset myocardial infarction**	*DNMT3A* (*OR 1.4*) (24)
	*TET2* (*OR 8.3*) (24)
	*ASXL1* (24)
	*JAK2 ^V617F^* (24)
**Coronary heart disease**	*DNMT3A* (*HR 1.7*) (24)
	*TET2* (*HR 1.9*) (24)
	*ASXL1* (*HR 2.0*) (24)
	*JAK2^V617F^* (*HR 12.1*) (24)
**Heart failure**	*DNMT3A* (51, 52)
	*TET2* (51–53)
	*ASXL1* (52, 53)
	*JAK2^V617F^* (53)
	*TP53* (54)
	*PPM1D* (54)
**Hemorrhagic, total, or ischemic stroke**	*DNMT3A* (55, 56)
	*TET2* (55–57)
	*ASXL1* (55, 57)
	*JAK2^V617F^* (58)
	*SRSF2* (57)
	*PPM1D* (57)
**Aortic stenosis**	*DNMT3A* (59, 60)
	*TET2* (59, 60)
**Peripheral artery disease**	*TP53* (61)
**Venous thromboembolism**	*JAK2^V617F^* (62)
**Pulmonary hypertension**	*TET2* (63)
	*JAK2^V617F^* (64)

OR, Odds Ratio; HR, Hazard Ratio.

CHIP has been associated with cardiovascular atherosclerotic disease (CAD). Notably, the *TET2* gene mutation has been strongly linked to increased cardiovascular morbidity and mortality due to CAD (24, 46, 47). Mutations in *TET2* were reported to accelerate atherosclerosis through altered function of the NOD-like receptor 3 (NLRP3)/interleukin-1ß (IL-1ß) inflammasome in mutant monocytes/macrophages residing in the blood vessel wall (46, 65). Such dysregulation of macrophage gene expression leads to increased activation of inflammatory pathways, including enhanced IL-1ß production (65). The ensuing chronic inflammatory state promotes the formation of atherosclerotic plaques, thus increasing the risk of myocardial infarction (MI) and stroke (66). CHIP was also found to be independently associated with adverse outcomes in individuals with established CAD, particularly in cases with *TET2* and spliceosome *(SF3B1/SRSF2/U2AF1)* mutations (47). The *JAK2^V617F^* mutation, known for promoting the proliferation of mature myeloid cells (48), has also been linked to increased AIM2 inflammasome activation and IL-1ß production (49), and with increased neutrophil cellular trap formation, thereby reportedly increasing the risk of atherosclerotic thrombosis in myeloproliferative neoplasms (MPNs) (62). An elevated burden of inflammatory macrophages within atherosclerotic lesions is associated with necrotic core formation and potential plaque instability (49). Inhibition of IL-1ß has shown promise in mitigating these effects (49, 50). Recently, Rauch and colleagues demonstrated that the loss of *DNMT3A* mimics the effects observed in the absence of *TET2 (* 45). This was evidenced by a consistent macrophage phenotype characterized by significant upregulation of CXCL1, CXCL2, CXCL3, and IL-6, ultimately contributing to the promotion of accelerated atherosclerosis (45).

The presence of CHIP has been associated with a 1.9-fold increase of coronary heart disease (CHD) (24). The risk of CHD is significantly increased in carriers of *DNMT3A, TET2*, and *ASXL1* CHIP mutations (1.7 to 2.0-fold increase), while *JAK2^V617^* CHIP carriers face an even higher risk of 12.1-fold elevation (24). *DNMT3A* is linked to elevated coronary-artery calcification (CAC) (24), highlighting its role in atherosclerotic burden. Notably, individuals with CHIP and atherosclerosis or CHD often lack traditional risk factors such as hypercholesterolemia, type 2 diabetes (T2D), hypertension, or smoking (67, 68). This suggests that the association between CHIP and CHD is primarily driven by an increased atherosclerotic burden, rather than other factors that may cause MI (24). This is in line with the finding that atherosclerosis is mainly driven by interactions between clonal monocytes-macrophages and the endothelium but also increased expression of pro-inflammatory genes such as IL-1ß and IL-6 (24, 46, 69, 70). Both atherosclerosis and coronary events follow a dose-response pattern with increasing size of the mutated clone (24). Mouse models using animals prone to hypercholesterolemia and carrying *TET2* mutations showed a marked increase in atherosclerotic lesions in the aortic root and aorta (24). These mice exhibited increased expression of several genes of the CXC chemokine gene cluster, including CXCL1, CXCL2, CXCL3, and Pf4, and classic proinflammatory cytokine genes such as IL-1ß and IL-6, which contribute to atherosclerosis (24). Additionally, these *TET2*-deficient mice displayed significantly larger plaque size (24, 46), increased atherosclerotic lesion area (24), xanthoma development in the spleen and middle ear (24), foam-cell accumulation and glomerulosclerosis in the kidney (24), and inflammatory infiltrates in the liver and lung (24).

CHIP is associated with a 4-fold increased risk of early-onset (before the age of 50 years) MI, combined with significant enrichment of mutations in *DNMT3A*, *TET2*, *JAK2*, and *ASXL1 (* 24). In patients with both ischemic and non-ischemic heart failure (HF), the presence of CHIP is linked to rapid disease progression and unfavorable overall survival (51, 71). The risk of cardiac death related to CHIP is dependent on the size of the clone, with larger clonal amplification (VAF >10%) correlating with a higher risk of HF (53). CHIP is generally associated with HF risk factors and biomarkers and is specifically associated with HF in individuals below 65 years of age (72). Mutations in *ASXL1*, *TET2*, and *JAK2* have emerged as new risk factors for HF, and are significantly associated with rapid HF progression, with odds ratios of 1.58x, 1.59x and 2.5x, respectively (52, 53). Also, DDR genes like *TP53* and *PPM1D* seem to be risk factors for HF (54). In studies of patients with chronic HF, approximately 18.5% exhibited 47 mutations with a VAF ≥ 2%, most commonly *DNMT3A* and *TET2* mutations, which were associated with worse long-term clinical outcomes, including death and rehospitalization (51, 52). Importantly, CHIP increased the risk of HF by 25%, independent of traditional CVD risk factors (53). Hematopoietic/myeloid dysfunction has been shown to contribute to pathological cardiac remodeling in HF mouse models with gene mutations in *TET2* and *DNMT3A (* 65, 73), suggesting that *TET2*-CH as well as *DNMT3A*-CH may generally play a role in altered heart remodeling and HF. Furthermore, *DNMT3A* and *JAK2* mutations, now known to promote atherosclerosis and HF, drive the proliferation of bone marrow cells with proinflammatory characteristics (24, 46, 53, 65, 73, 74). Recently, Abplanalp and colleagues conducted an in-depth exploration of the cell-intrinsic effects of CHIP in HF (75). Their findings revealed that *DNMT3A*-mutant monocytes exhibited elevated expression of genes associated with inflammation (*CCR2*) and phagocytosis (*CALR, CYBA*) *(* 75). Additionally, CD4^+^ T cells and NK cells displayed heightened activation signatures and effector functions (75). CD4^+^ T cells demonstrated upregulation of markers indicative of T cell effector and killing function, such as *LYZ* and *CXCL8*, along with increased expression of drivers of proliferation (*SELENOF*) and markers of T cell differentiation (*SLAMF6*) (75). Similarly, CD4^+^ NK cells exhibited increased expression of the important lysozyme molecule, *LYZ (* 75). Importantly, they demonstrated that *DNMT3A*-mutant macrophages can indirectly promote pro-hypertrophic activity in wild-type macrophages through paracrine signaling (75).

Individuals with CHIP have an increased risk of hemorrhagic or ischemic stroke (55, 57). The analysis of clonal dynamics following an ischemic stroke revealed that clones with a VAF of 1% have an estimated doubling time of approximately 8 years on average (57). Notably, *SRSF2*-mutated clones displayed the most robust expansion characteristics, doubling in size every 2.8 years (57). Therefore, individuals harboring *SRSF2*-mutated clones are at the greatest risk of developing a hematologic malignancy following an ischemic stroke. Furthermore, *TET2* mutations are highly enriched in patients with ischemic stroke (57), suggesting a central role of *TET2*-driven CH in stroke pathogenesis (57). Other studies have identified *DNMT3A* (30.0%) and *TET2* (11.4%) as the most common mutations in individuals with CHIP who experienced a first-ever acute ischemic stroke, and CHIP was specifically associated with recurrent stroke in patients with hyperinflammation (56). *TET2* showed the strongest association with total and ischemic strokes, while both *DNMT3A* and *TET2* were associated with an increased risk of hemorrhagic stroke (55). Moreover, mutations in *ASXL1* and *PPM1D* have been associated with stroke, too (55, 57). More recently, the *JAK2^V617F^* mutation has also been associated with ischemic stroke, with a prevalence of 11.3% (58). Patients who experienced an ischemic stroke and had CH mutations displayed elevated levels of Interferon-γ (IFN-γ) (57). In addition, the presence of *TET2* mutations was associated with elevated IL-1ß levels (57). Furthermore, there was a notable increase in white matter lesion load, which showed correlations with patient age and clone size (57).

CHIP has also been implicated in poor outcomes following transcatheter aortic valve implantation (TAVI) in patients with calcific aortic stenosis (76). In such patients with severe degenerative aortic valve stenosis, *DNMT3A* and *TET2* mutations again played a significant role in promoting higher monocyte expression of inflammatory mediators such as IL-1ß, IL-6, and NLRP3 (59, 60, 77, 78). Furthermore, CHIP has been associated with peripheral artery disease (*TP53*) (61), venous thromboembolism (*JAK2^V617F^*) (62) and pulmonary hypertension (*TET2*, *JAK2^V617F^*) *(* 63, 64).

## Etiology of CHIP and associated diseases

A variety of factors have been reported to contribute to the development of CHIP and the diseases that are promoted or aggravated by CHIP. Such risk factors are listed in **Table 2**.

**Table 2:** CHIP, risk factors and associated diseases.

Risk factor or associated disease	Related CHIP-mutations
**Germline variation**	*TERT* (32, 79–81)*, SH2B3* (79)*, TET2* (79, 80)*, ATM* (79)*, CHEK2* (79, 80)*, PINT* (79)*, GF11B* (79)*, DNMT3A* (80, 82)*, JAK2^V617F^* (83)*, BRCA1/2* (41, 84)*, BARD1* (84)*, TP53* (84)
**Aging**	*TERT* (32, 80, 85–87)
**Obesity, insulin resistance, Type 2 Diabetes**	*DNMT3A* (88)*, TET2* (89, 90)
**Birth weight**	*DNMT3A* (91)
**Sleep deprivation**	*TET2* (92)
**Unhealthy diet**	*DNMT3A* (93)*, TET2* (93)*, ASXL1* (93)
**COPD**	*DNMT3A* (94, 95)*, TET2* (94)
**Smoking**	*ASXL1* (38, 94, 96), *TET2* (94)
**Premature menopause**	*DNMT3A* (97)
**Systemic sclerosis**	*DNMT3A* (98)
**Rheumatoid arthritis**	*DNMT3A* (99)*, TET2* (99)
**Ulcerative colitis**	*DNMT3A* (100)*, PPM1D* (100)
**Chronic liver disease**	*TET2* (101)
**Chronic kidney disease**	*DNMT3A, TET2* (102)
**Osteoporosis**	*DNMT3A (* 103)
**Gout**	*TET2 (* 104)
**Chronic mycobacterial infection**	*DNMT3A* (105)
**HIV**	*DNMT3A* (106, 107)*, TET2* (107)*, ASXL1* (106–108)
**SARS-CoV-2**	*PPM1D* (109)
**Clostridial or streptococcus/enterococcus**	(109)
**Solid tumor malignancies**	*DNMT3A, TP53, TET2, PPM1D, ATM, ASXL1, CHEK2, JAK2, CBL, SF3B1, IDH2, U2AF1, IDH1*, and *RUNX1* (35)
**Cancer treatment**	*TP53* (39, 110–114)*, PPM1D* (38, 39, 110–117 *), CHEK2* (38, 110–112, 117), *DNMT3A* (118)*, TET2* (118)*, ASXL1* (118)
**Autologous SCT**	*DNMT3A (* 119, 120)*, TET2* (119)*, TP53* (119)*, ASXL1* (119)*, PPM1D* (119, 120)
**Allogeneic SCT**	*TP53, DDX41* (121)
**CAR-T cell therapy**	*DNMT3A* (122, 123)*, TET2* (122, 123)

COPD, Chronic obstructive pulmonary disease; HIV, Human immunodeficiency virus; SARS-CoV-2, severe acute respiratory syndrome coronavirus type 2; SCT, Stem cell transplantation; CAR-T, Chimeric Antigen Receptor (CAR)-T cell therapy.

The development of CHIP and its association with various diseases is influenced by several risk factors. Germline variation in genes such as *TERT*, *SH2B3*, *TET2*, *ATM*, *CHEK2*, *PINT*, and *GF11B* plays a significant role in predisposing individuals to the emergence of CHIP (79). The *TERT* locus, in particular, harbors the most important risk allele for CHIP (80, 81), since germline deletion of intron 3 results in decreased leukocyte telomere length (LTL) (32). Altogether, four gene loci have been identified that are variably associated with an increased susceptibility to *TET2*-CHIP (80), namely *TERT, CHEK, TET2*, and the *TCL1A* locus, which also results in an elevated risk of *DNMT3A*-CHIP *(* 80). In contrast, missense variants in *PARP1* and *LY75* are associated with lower CHIP incidence (81). The driver mutation *JAK2^V617^* can be acquired as early as *in utero* or childhood, followed by lifelong clonal evolution eventually leading to MPNs (83). Acquisition of a *DNMT3A* mutation has also been detected to occur *in utero* or childhood (82). Another study identified cancer susceptibility germline mutations in the DDR genes *BRCA1, BRCA2, BARD1* and *TP53* in patients with t-MNs (84). The reported association of g*BRCA1/2* mutation status and increased t-MN incidence is probably indirect, owing to more extensive platinum-based chemotherapy, which increased the likelihood of accumulating CH-associated gene mutations in *PPM1D* and *TP53 (* 41). Studies of CHIP prevalence have revealed interesting patterns regarding race, ethnicity, and ancestry. There is a modestly lower prevalence of CHIP in Hispanics and East Asians compared to other populations (24, 124). Similarly, previous studies showed that the prevalence of AML is lower in East Asian populations than in Caucasian populations (125). CHIP may thus be more common in the Western population. However, a particular germline variant in *TET2*, rs144418061, which is associated with an increased risk of CHIP, is found exclusively in individuals of African descent (80), highlighting the importance of considering ancestral backgrounds in understanding CHIP prevalence.

Aging is a major factor in the accumulation of somatic mutations in HSCs due to cumulative DNA damage (126), and patients with inherited telomeropathies have a higher prevalence of CHIP (85), with common variants at the *TERT* gene (80) (telomere brink hypothesis (32, 86, 87, 127)). Recently, familial CH was reported in a long telomere syndrome (128). The authors suggested that delayed telomere attrition or even telomere lengthening over time allowed for the expansion of CHIP clones by enabling multiple cell divisions while avoiding cellular senescence (128). Hotspot mutations *DNMT3A* and *TET2* were most common (128).

CHIP is furthermore associated with obesity, insulin resistance, and T2D. Obesity-related insulin resistance is associated with a higher incidence of *TET2*-CHIP *(* 89). In general, diabetic patients exhibit 1.3-fold increased odds of CHIP (5). In mice, *TET2*-driven CHIP leads to an increased expression of IL-1ß in white adipose tissue, exacerbating age- and obesity-related insulin-resistance and hyperglycemia (89). This sets off a positive feedback loop (89, 90), promoting HSC self-renewal and inflammation progression, ultimately resulting in the loss of ß-cell mass in T2D (89, 129) which, in the end, would be responsible for the pathogenesis of diabetes and atherosclerosis (90). *DNMT3A* mutations are also more frequent in adipose tissue-derived macrophages in mice fed a high-fat diet (88). Moreover, a recent study demonstrated that birth weight is associated with CHIP and cardiovascular outcomes in adulthood (91). A 1-kg increase in birth weight was associated with a 3% increased risk of CHIP, mainly driven by a stronger association observed between birth weight and *DNMT3A*-CHIP *(* 91). Sleep deprivation enhances the incidence of CVD, diabetes, obesity, and cancer (130). Sleep-deprived mice have increased atherosclerotic lesions, along with systemic monocytosis and neutrophilia (130). When sleep is fragmented, *Tet2^-/-^* Ldlr^-/-^ mice show a 1.6-fold increase in the emergence of CHIP (92). Addiction and psychiatric diseases are also associated with CHIP, potentially linked to the critical component of sleep fragmentation (32). Unhealthy diet, characterized by highly processed food, red meat, added salt, and low intake of fruits and vegetables, is associated with a higher incidence of CHIP, CVD, and mortality (93). *DNMT3A*, *TET2*, and *ASXL1* mutations were associated with unhealthy diet, although no statistically significant differences were observed between the distributions of CHIP-associated genes across dietary classes (93). However, some studies show no direct associations between diet and CHIP (131), possibly attributable to variations in study populations, measurement errors, and other factors that require further investigation (132).

Smoking consistently associates with CHIP (6, 32, 96), and individuals who never smoked have a decreased risk of developing CHIP (131). Current and past smokers show a higher frequency of *ASXL1* mutations (38, 94, 96). Tobacco smoke has also been found to promote the expansion of *TET2*-mutated blood cells, potentially acting as a contributing factor to chronic obstructive pulmonary disease (COPD) (94). COPD is associated with CHIP (94), with *DNMT3A* mutations most commonly involved (95). The risk factors for COPD, such as smoking and aging, are also important risk factors for CHIP (94). Individuals with CHIP have a 1.6-fold and 2.2-fold increased risk of moderate and very severe COPD, respectively (94). In mouse models, the absence of *TET2* has been linked to increased lung infiltration of immune cells, elevated IFN-γ levels, reduced Transforming growth factor ß (TGF-ß) signaling in immune cells, and accelerated development of emphysema in the lungs (94). In COPD patients, defects in *DNMT3A* in macrophages have been associated with increased production of IL-6 and tumor necrosis factor (TNF)-α, as well as global hypomethylation in the genome (95). To date, it is uncertain in which causal relationship CHIP and COPD are related to each other, however, based on previous findings, it is likely that the presence of somatic mutations such as CHIP contributes to the development and exacerbation of COPD (94).

Premature menopause (PM), with an odds ratio of 1.40, is linked to an increased risk of CHIP (97). PM is furthermore associated with a higher incidence of CAD (HR 1.36), with a stronger association observed in cases of natural PM (97). *DNMT3A* is the only candidate driver mutation associated with PM-related CHIP (97).

CHIP is also associated with various chronic inflammatory conditions. Patients with systemic sclerosis (SS) have an elevated prevalence of CHIP, with 25% of individuals affected compared to only 4% in the control cohort without SS (98). Notably, among the CHIP cases in SS, *DNMT3A* was the most commonly mutated gene (98). Similarly, rheumatoid arthritis (RA) patients have a 17% higher prevalence of CHIP, which increases to 25% in individuals aged 70-79 years, with *DNMT3A* and *TET2* mutations being the most frequent (99). Patients with ulcerative colitis (UC), an inflammatory bowel disease (IBD) characterized by T-cell infiltration in the colon and overproduction of TNF-α and IFN-γ, also have an association with CHIP (100). These patients are at an increased risk of ischemic heart disease (133–135), with *DNMT3A* and *PPM1D* mutations being the most common alterations (100, 136). Since patients with IBD are a high-risk population for developing CH, there is concern that clonal expansion induced by inflammation could potentially make IBD patients with CHIP more prone to develop MNs (136). Furthermore, IBD carriers carry elevated levels of Th17 cells and Th17 related cytokines which are important in the pathogenesis of mucosal damage in IBD (137), displaying a similar pathogenesis to *DNMT3A*-CHIP carriers with severe degenerative aortic stenosis (60). More recently, CHIP was associated with an increased prevalence and incidence of chronic liver disease (OR = 2.01), with CHIP individuals being more likely to show liver inflammation and fibrosis compared to those without CHIP (101). Similarly, mice transplanted with *TET2*-deficient HSCs had more severe liver inflammation and fibrosis (101). Another chronic disease associated with CHIP is chronic kidney disease, with gene mutations most commonly found in *DNMT3A* and *TET2 (* 102). CHIP is linked to kidney function decline and end-stage kidney disease in the general population, although the effect sizes were relatively modest (138). Moreover, it has been observed that impaired kidney function associated with CHIP is correlated with anemia in patients with advanced chronic kidney disease (102). Furthermore, research has demonstrated the association of *DNMT3A*-CHIP with an elevated occurrence of osteoporosis diagnoses and reduced bone marrow density (103). This phenomenon is driven by the expression of proinflammatory IL-20 from *DNMT3A*-mutant macrophages (103). Further, *TET2*-mutant CHIP is linked to an increased risk of incident **gout**. This association is supported by the observation of heightened NLRP3-independent IL-1ß secretion from *TET2* knockout macrophages in response to monosodium urate crystals *in vitro* (104), indicating a pathogenesis similar to that of gout. CHIP is associated with a broad range of chronic infections. Mice models have shown that chronic mycobacterial infection leads to *DNMT3A*-CHIP clone expansion through an IFN-γ-dependent mechanism (105). Moreover, HIV patients have a twofold higher prevalence of CHIP (108), and HIV is associated with a higher risk of MDS (106). The Swiss HIV cohort study found a greater prevalence of *ASXL1* mutations (108), while the ARCHIVE study revealed a higher prevalence of *DNMT3A* (48.5%), *TET2* (20.5%), and *ASXL1* (11.4%) mutations in HIV patients (107). *ASXL1* and *DNMT3A* mutations are frequently detected in HIV patients with MDS (106). Given the association of CHIP with increased cardiac risk, it is worth exploring whether CHIP could explain the elevated risk of CVD in HIV patients (139). Moreover, *PPM1D*-CHIP carriers have been observed to experience a more severe course of disease following SARS-CoV-2 infection (109), and CHIP is associated with an increased risk of clostridial or streptococcal/enterococcal infections (109).

CHIP is common among patients with solid tumors, as summarized by Marshall and colleagues (35). Associations exist between CHIP and several types of cancer, including bladder tumors (38, 140, 141), breast cancer (38, 140–143), colorectal cancer (38, 140, 142, 144, 145), endometrial cancer (38, 140), esophagogastric cancer (38, 140, 143), head and neck cancer (38, 140, 143), kidney cancer (38, 140, 141), lung cancer (38, 140–143), melanoma (38, 140, 141), ovarian cancer (38, 41, 140, 143), pancreatic cancer (38, 140–143), prostate cancer (38, 140–142, 146), and thyroid cancer (38, 140, 141, 143). Across those malignancies, mutations in the genes *DNMT3A, TP53, TET2, PPM1D, ATM, ASXL1, CHEK2, JAK2, CBL, SF3B1, IDH2, U2AF1, IDH1*, and *RUNX1* were the most common (35). The presence of CHIP in these patients suggests a potential link between CH and the development or progression of solid tumors. CHIP-associated mutations may play a contributory role in the initiation and progression of certain cancers by promoting increased inflammatory activity, DNA damage, and other pertinent cellular alterations.

Finally, cancer treatment is strongly associated with CHIP. There is compelling evidence that CH mutations may drive the transformation into t-MNs (38). Concerning the progression of CH to t-MNs, it has been observed that mutations are differentially selected based on various drug exposures. Therefore, it is essential to identify patients who exhibit risk factors associated with the development of t-MN (38). The highest CHIP prevalence is observed in patients who have undergone external beam radiation therapy, cytotoxic chemotherapy (particularly with topoisomerase II inhibitors, anthracyclines, or carboplatin), and radionuclide therapy (38). Furthermore, ambient exposure to radiation is associated with CHIP, as exemplified by the NASA Twin Study where two astronauts exhibited CHIP nearly two decades earlier than the mean age and displayed more significant shifts in clone size compared to age-matched controls or radiotherapy patients (147). It has been shown that ionizing radiation, topoisomerase II inhibitors, and cisplatin select for mutations in DDR genes *TP53*, *PPM1D*, and *CHEK2 (* 38, 110–112). Particularly the chemotherapy agents cisplatin, etoposide, and doxorubicin selectively promote the growth of *TP53*- and *PPM1D*-mutated CHIP clones that are resistant to chemotherapeutic agents (110). Those mutations are more commonly detected in therapy-related acute myeloid leukemia (t-AML) than *de novo* AML (110). Similarly, *CHEK2*-mutant blood cells can expand under therapy with topoisomerase inhibitors, leading to secondary MN, such as t-AML or secondary MDS (38). Clones with lower VAF can be detected in the blood even before t-AML diagnosis and prior to chemotherapy (112, 148). *PPM1D* mutations are most frequently observed in secondary therapy-related myelodysplastic syndromes (t-MDS) (110, 115) and in patients with bronchial (116) and ovarian carcinoma (113) after chemotherapy. In patients with ovarian carcinoma, *TP53* mutations may also be found after chemotherapy (113). Presumably, mutations in DDR genes confer a selective advantage to HSCs under the selective pressure exerted on the hematopoietic system, with certain cytotoxic classes including platinum, radionuclide therapy, and topoisomerase I/II inhibitors creating a particular advantage for DDR-mutant HSCs (38, 117). While *TP53* mutations provide a selective advantage to cells under cytotoxic stress through a dominant-negative effect, mutations in exon 6 of *PPM1D* lead to a truncated gene product lacking a C-terminal degradation domain, which prolongs the lifespan of the protein (114). *PPM1D*-mutated HSCs exhibit reduced apoptosis and increased resistance to cytotoxic chemotherapy, allowing the clones to expand under chemotherapy (110, 114). In contrast to DDR mutations, mutated DTA genes tend to foster proliferation and clonal expansion through mechanisms involving inflammation (118). A few cycles of chemotherapy are sufficient to cause significant expansion of a CH clone (149). Several risk factors have been identified for the development of t-MNs, including the number of mutations, clone size, presence of *TP53* and *PPM1D* mutations, spliceosome mutations, and exposure to PARP inhibitor therapy (38, 39). Using PARP inhibitors in tumors with homologous recombination deficiency due to germline mutations in *BRCA1/2* can provide a selective advantage for pre-existing *TP53*- and *PPM1D*-mutated clones, resulting in increased t-MN risk (39–41). This advantage is mainly attributed to the increased exposure to platinum-containing cytostatic drugs and PARP-inhibitors, such as rucaparib, used in high-grade ovarian cancer treatment (40, 41). CH-related alterations in *TP53* (most common), *ATM*, *GNAS*, and *JAK2* have been identified in cell-free DNA from patients with invasive glioma who underwent treatment with temozolomide, a mutagenic alkylating agent, in combination with radiation therapy (150). It is believed that in deep remission from the primary disease, a CHIP clone may persist and accumulate additional mutations, potentially leading to the development of a myeloid neoplasia (151).

In the context of cell therapies, such as autologous stem cell transplantation (SCT), allogeneic SCT, and Chimeric Antigen Receptor (CAR)-T cell therapy, an increased prevalence of CHIP has been observed. For example, SCT for Non-Hodgkin lymphoma is associated with a 30% incidence of CHIP (152). In autologous SCT, the prior exposure to cytotoxic agents used in conventional chemotherapeutic regimens can promote the occurrence of *de novo* mutations and also favor the selection and expansion of existing clones, with most common mutations observed in *DNMT3A, TET2, TP53, ASXL1*, and *PPM1D (* 119). Patients with single and comutated *DNMT3A-* and *PPM1D*-CH were found to have lower stem cell yield and delayed recovery of platelet count after autologous SCT (120), thus suggesting investigation for CHIP prior to autologous SCT may be plausible. In allogeneic SCT, which involves the transfer of HSCs from a foreign donor to the recipient, a rare complication known as donor cell leukemia (DCL) or secondary myeloid neoplasia can occur. The complication seems to result from the transfer of pre-existing donor CHIP to the recipient (153). It has been estimated that there are approximately 0.8% DCLs in Europe, with 28% of them being CHIP-related (153). However, it has also been demonstrated that the presence of donor *DNMT3A*-CH is associated with enhanced recipient survival due to a decreased risk of relapse and an expanded network of inflammatory cytokines in recipients (121). The risk of DCL in allogeneic hematopoietic cell transplantation is primarily driven by somatic mutations associated with MDS, particularly in genes like *TP53* or splicing factor mutations (121). In addition, there is a risk of DCL associated with germline predisposition in donors, particularly when mutations in the *DDX41* gene are present (121).

Although it appears that CHIP is generally associated with unfavorable outcomes, there are at least two exceptions. In the context of CAR-T cell therapy, which exclusively utilizes autologous preparations, the presence of CHIP can influence the treatment outcome beneficially. CHIP can contribute to increased inflammatory reactions and altered interactions between immune cells, potentially affecting the efficacy and behavior of the transfected immune cells (24, 124, 154). Mutations in genes such as *TET2* and *DNMT3A*, which are commonly associated with CHIP, may thus have implications for CAR-T cell therapy by enhancing antitumor activity (122, 123). Overall, there is a consistently high prevalence of CHIP prior to CAR-T cell therapy (ranging from 34% to 48%) (155). This high prevalence may be attributed to the intensive pretreatment received by these patients, contributing to frequent clonal progression in the long term after CAR-T cell therapy, which is relevant to the development of t-MNs (155). Recently, CHIP was shown to attenuate the risk of Alzheimer’s Disease (AD). A higher VAF was significantly associated with protection against AD when modeled as a continuous variable (156). CHIP-associated mutations were found in brain samples (156, 157), presumably due to infiltration of the brain by marrow-derived mutant myeloid cells that have adopted a microglial-like phenotype (156). These mutant cells residing in the brain may ameliorate neurodegenerative disorders due to their superior phagocytic activity.

**Table 3** summarizes CHIP-associated genes, presumed mechanisms of action, and related medical conditions. The listed mechanisms reflect our current knowledge rather than a comprehensive understanding of each gene’s function and its impact on CHIP-associated outcomes.

**Table 3:** Frequently mutated CHIP genes and associated mechanisms.

Gene	Function of the gene	Perturbed gene function	Putative pathologic mechanism	Associated medical conditions
***DNMT3A***	Epigenetic regulation of gene expression (DNA *de novo* methylation)	Altered DNA methylation and epigenetic dysregulation	NLRP3 inflammasome activation (158)	CAD (45), Early-onset MI (24), incident CHD (24), chronic HF (51, 52), Ischemic (56)/hemorrhagic (55) stroke, aortic stenosis (59, 60), COPD (94, 95), PM (97), SS (98), RA (99), UC (100), chronic kidney disease (102), osteoporosis (103), chronic mycobacterial infection (105), HIV (107), MDS-HIV (106), solid tumor malignancies (35)
			↑ Th17/Treg ratio (60)	
			↑ TNF-α (95), IL-1ß (158), IL-6 (45, 158), IL-20 (103)	
			↑ CXCL1 (45), CXCL2 (45, 158), CXCL3 (45)	
			↑ CCL3, CCL4, resistin (158)	
			↑ CCR2, CALR, CYBA, LYZ, CXCL8, SELENOF, SLAMF6 (75)	
***TET2***	Epigenetic regulation of gene expression (DNA demethylation)	Altered DNA methylation and epigenetic dysregulation	NLRP3 inflammasome activation (46, 65, 73)	CAD (24, 46, 47), early-onset MI (24), incident CHD (24), chronic HF (51–53), ischemic (55, 57)/total (55)/hemorrhagic (55) stroke, aortic stenosis (59, 60), pulmonary hypertension (63), diabetes (89), COPD (94), RA (99), chronic liver disease (101), chronic kidney disease (102), gout (104), HIV (107), solid tumor malignancies (35)
			↑ Nonclassical monocytes (60)	
			↑ IL-1ß (24, 57), IL-6 (24)	
			↑ CXCL1, CXCL2, CXCL3, Pf4 (24)	
			↑ IFN-γ levels (94)	
			↓ TGF-ß signaling (94)	
			STING activation (159)	
***ASXL1***	Epigenetic regulation of gene expression (Mono ubiquitination of H2A)	Aberrant chromatin remodeling	Akt/mTOR pathway activation (160)	Early-onset MI (24), incident CHD (24), HF (52, 53), stroke (55, 57), HIV (106–108), MDS-HIV (106), solid tumor malignancies (35)
			↓ T-cell infiltration at tumor sites (161)	
			↑ programmed death receptor-1 (PD-1) in CD8^+^ T cells (161)	
			T cell dysregulation: aberrant intrathymic T-cell development, ↓ CD4/CD8 ratio, naïve-memory imbalance in peripheral T cells (161)	
***JAK2***	Tyrosine kinase	Constitutive activation of JAK-STAT signaling pathway	AIM2 inflammasome activation (34)	CAD (48–50), early-onset MI (24), incident CHD (24), HF (53), ischemic stroke (58), venous thromboembolism (62), pulmonary hypertension (64), solid tumor malignancies (35)
			↑ neutrophil extracellular traps (62)	
			↑ burden of inflammatory macrophages within atherosclerotic lesions, necrotic core formation, and potential plaque instability (34)	
			↑ IL-1ß (34), IL-18 (80), ↑ IL-6 (80)	
***TP53***	DNA damage repair, Cell cycle control	Disruption of cell cycle control and DNA damage response resulting in selective growth advantage to affected cells	↑ H3K27 tri-methylation of genes regulating differentiation and HSC self-renewal (162)	HF (54), peripheral artery disease (61), solid tumor malignancies (35), t-AML (110)
			↑ reactive oxygen species (163)	
***PPM1D***	DNA damage repair, Cell cycle control	protein truncation in exon 6 resulting in impaired TP53 function, enhanced cell survival and resistance to DNA damage	↑ reactive oxygen species (163)	HF (54), stroke (57), UC (100), SARS-CoV-2 (109), solid tumor malignancies (35), t-AML (110), t-MDS (110, 115)
			↑ IL-1ß, IL-18 (163)	
***CHEK2***	DNA damage repair, cell cycle control	Enhanced cell survival and resistance to DNA damage	↑ HSC self-renewal (164)	solid tumor malignancies (35), t-AML (38), secondary MDS (38)
***SF3B1***	Splicing	Splicing factor dysfunction	↑ IL-18 (80)	CAD (47), MDS (31, 36), solid tumor malignancies (35)
***SRSF2***	Splicing	Splicing defects	↓ EZH2 (165)	CAD (47), stroke (57), MDS (31, 36)
***U2AF1***	Splicing	Splicing factor dysfunction	↓ EZH2 (165)	CAD (47), MDS (31, 36), solid tumor malignancies (35)

NLRP3, NOD-like receptor 3; TNF, Tumor necrosis factor; IL, Interleukin; IFN-γ, Interferon gamma; TGF-ß, Transforming growth factor ß; CHD, Coronary heart disease; MI, Myocardial infarction; HIV, Human immunodeficiency virus; (t-)MDS, (therapy-related) myelodysplastic syndromes; COPD, chronic obstructive pulmonary disease; PM, premature menopause; HF, heart failure; SS, systemic sclerosis; RA, rheumatoid arthritis; UC, ulcerative colitis; CAD, cardiovascular atherosclerotic disease; CHD, coronary heart disease; MPNs, myelodysplastic syndromes; (t-)AML, (therapy-related) acute myeloid leukemia.

The symbol ↑ means “increase”, whereas the symbol ↓ means “decrease”.

## Future perspectives and clinical implications

Research on CHIP has already yielded significant insights, but many questions remain. A better knowledge of the impact of genetic mutations on cellular function could help to develop new diagnostic and therapeutic approaches. Identifying individuals with CHIP is expected to enable more precise risk assessment for hematologic disorders and cardiovascular complications, potentially leading to improved prevention and treatment strategies. Several studies have explored therapeutic approaches for cardiovascular risks related to CHIP (s. **Table 4**).

**Table 4:** Current therapeutical approaches for CHIP.

Therapeutical approach	Target
**Presence of the inhibitory IL-6 receptor gene variant IL6Rp.Asp358Ala (** 124 ) (*)	Inhibition of IL-6 receptor in *DNMT3A*- and *TET2*-CHIP carriers → CVD risk reduction by 50%
**Canakinumab (CANTOS trial) (** 166, 167 ) (*)	IL-1ß blockade in *TET2*-CHIP carriers → reduction of major cardiovascular events
**Ziltivekimab (** 168 ) (*)	IL-6 inhibition in patients at high atherosclerotic risk → normalized C-reactive protein and biomarkers associated with atherosclerosis; fibrinogen and lipoprotein(a)
**Colchicine (COLCOT study) (** 169 ) (*)	IL-1 and IL-8 blockade in patients post-MI → significantly lower risk of ischemic cardiovascular events
**Anakinra (** 49 ) (*)	IL-1 receptor blockade in *JAK2*-CHIP carriers → reduced atherosclerotic plaque instability with normalized macrophage proliferation and density in early atherosclerotic plaques, and reduced core necrosis, increased cap thickness in advanced atherosclerotic plaques
**MCC950 (** 46 ) (*)	Pharmacologic inhibition of the NLRP3 inflammasome in *TET2*-deficient mice → 50% decrease in aortic atherosclerotic plaque size
**Vitamin C (** 170 ) (**)	TET2 activation and restoration in *TET2*-deficient mice → reversion of aberrant HSC self-renewal
**Ruxolitinib (** 49, 62 ) (**)	*JAK2* inhibition in *JAK2^V617^ mice* → reduced abnormal NET formation, reduced deep vein thrombosis, and reduced IL-18 circulating levels
**Fedratinib (** 171 ) (**)	*JAK2* inhibition in *Apoe^-/-^* mice → suppressed myelopoiesis and atherosclerosis development
**Rapamycin (** 160 ) (**)	mTOR inhibition in *ASXL1*-deficient *mice* → blocked expansion of *ASXL1*-mutated HSCs
Metformin (**) ( 172)	lowering of glucose levels in diabetic mice → rescued *TET2* activity

IL, Interleukin; CVD, Cardiovascular Disease; NLRP3, NOD-like receptor 3; HSC, Hematopoietic stem cell; MI, Myocardial infarction; NET, Neutrophil extracellular traps. * Inhibition of inflammatory mediators. ** Targeting specific candidate driver mutations.

Inhibition of inflammatory mediators has been a focus in these studies. For example, the CANTOS clinical trial demonstrated that blocking IL-1ß (an upstream mediator of IL-6) with canakinumab resulted in reduced major adverse cardiovascular events in high-risk atherosclerosis patients (167). The reduction in events was more pronounced in *TET2*-CHIP patients (166). A phase-2 study is currently being conducted in Leipzig, Germany, testing the efficacy and safety of canakinumab for the treatment of anemia in low-risk MDS patients (ClinicalTrials.gov Identifier: NCT05237713). Another study used ziltivekimab for IL-6 inhibition in patients at high atherosclerotic risk and achieved normalized expression of C-reactive protein and biomarkers associated with atherosclerosis, such as fibrinogen and lipoprotein(a) (168). Interestingly, the presence of the inhibitory IL-6 receptor gene variant IL6R p.Asp.358 Ala reduced CVD risk by 50% in *DNMT3A*- and *TET2*-CHIP carriers (124). Non-selective IL-1 receptor blockade with anakinra and targeted IL-1ß blockade in a *JAK2*-CHIP model resulted in decreased atherosclerotic plaque instability, normalized macrophage proliferation and density in early plaques, and reduced core necrosis and increased cap thickness in advanced plaques (49). The COLCOT study explored modulating IL-1 and IL-8 using colchicine in patients recruited within 30 days post-MI, thereby significantly lowering risk of ischemic cardiovascular events (169). Pharmacologic inhibition of the NLRP3 inflammasome with MCC950 in *TET2*-deficient mice led to a 50% decrease in aortic atherosclerotic plaque size, which was greater than the nonsignificant reduction observed in wildtype mice (46). Targeted inhibition of the AIM2 inflammasome, which is overactive in *JAK2*-mutant CHIP, may offer similar benefits (49).

Further investigations have focused on targeting specific driver mutations in CHIP. For example, vitamin C metabolites that activate *TET2* and mimic the restoration of *TET2* via elevated 5-hydroxymethycytosine formation in *TET2*-deficient mice showed potential as a therapy for *TET2*-CHIP carriers (170). Treatment with the *JAK2* inhibitor ruxolitinib reduced abnormal NET formation (62), deep vein thrombosis (62), and circulating levels of IL-18 (49) in *JAK2^V617^ mice*. *JAK2* inhibition with fedratinib in *Apoe* ^-/-^ mice suppressed myelopoiesis and the development of atherosclerosis (171). Rapamycin treatment has been shown to block expansion of *ASXL1*-mutated HSCs through mTOR inhibition (160). The heightened self-renewal and neoplastic transformation observed in *TET2*-mutated HSPCs has recently been demonstrated to result from the activation of the cyclic guanosine monophosphate-adenosine monophosphate synthase (cGAS)-stimulator of interferon genes (STING) pathway (159). Hence, targeting the STING pathway could represent a promising intervention strategy for relevant hematopoietic diseases. Finally, *TET2* activity could be restored in diabetic mice by lowering blood glucose levels with metformin (172), suggesting that this may also hold promise for mitigating CVD risk in patients with *TET2*-driven CHIP.

Overall, some therapeutic approaches are emerging that mainly focus on inhibition of inflammatory pathways and restoration of gene function. However, further research is needed to confirm the efficacy and safety of such treatments in individuals with CHIP.

## CHIP detection and risk management

The prevalence of CHIP depends on the age of the study cohort and the sensitivity of the DNA sequencing method (26). Currently, the threshold for CHIP is a VAF ≥2%, which means that a heterozygous somatic mutation in a leukemia-associated gene is present in ≥4% of all blood cells (5). Since there is generally no screening for CHIP in routine clinical practice, CHIP is often an incidental finding during tumor diagnostics, for example, when DNA from blood cells serves as a normal control, or in the context of genetic workup for other disease (149). Regarding molecular tumor diagnostics, it is important to note that, as long as a normal wildtype finding in the blood is not available, CH-related driver mutations detected in tumor tissue should not be interpreted as tumor mutations (149). It has been shown that mutations in CH-associated genes were detectable in 65% of solid tumors, of which 8% were CH mutations instead of tumor mutations, with *DNMT3A* most common (173). This interference can be resolved by parallel sequencing of tumor material and DNA from peripheral blood (149).

The most sensitive technique, i.e. ‘error corrected sequencing’ (ECS) can identify point mutations and small indels from adult blood and marrow samples at a VAF as low as 0.01% (26, 174). ‘Targeted sequencing’ looks at a specific selection of genes, mostly through gene panel sequencing using next generation sequencing (NGS), detecting clones with a VAF ≥0.5% (26, 175). Targeted sequencing provides greater sequencing depth and sensitivity, and therefore detects more people with CH (26), than whole exome (WES) (5, 6) or whole genome (WGS) sequencing (32), which typically have a much lower depth of coverage. WGS covers the entire genome but is relatively insensitive to smaller clones, detecting clones with a VAF ≥7% (26). WES covers only the protein-coding regions of the genome but is somewhat more sensitive to smaller clones, detecting clones with a VAF around 3% or higher (26). Currently, the biological significance of very small clones is unclear (175–177).

Regarding the future management of people with CHIP, setting up ‘CHIP clinics’ is an important initiative. Here, individuals with certain risk factors for CHIP will be offered adequate screening, and individuals who are already diagnosed with CHIP will receive counseling and, if possible, a plan for intervention, considering individual lifestyle and environmental influences (178). For that purpose, ideally, hematologists and cardiologists should cooperate with hepatologists, geneticists, psychologists, and nutritionists in an interdisciplinary consultative clinic (178). Comprehensive screening for CHIP mutations and identification of potential correlations with clinical patterns will facilitate consultation tailored to patients’ individual needs and will also support further research into CHIP.

However, the absence of established guidelines for “CHIP consultation” suggests that the way forward will not be easy. There is pronounced variability regarding the health effects of CHIP mutations, depending on genes affected, VAFs, dynamics of clonal expansion, and interaction between multiple genetic aberrations. This precludes simple algorithms for counseling.

In terms of hematological risk management, CHIP consultation is particularly relevant for patients carrying mutations linked to an increased risk of hematological malignancy, such as driver mutations for MNs, especially *IDH1/IDH2*, *TP53*, or spliceosome genes (179). The Clonal Hematopoiesis Risk Score (CHRS) is a prognostic tool to assess the risk of malignancy in individuals with CHIP or CCUS. It helps to identify a minority of high-risk CHIP/CCUS cases among the majority of cases with minimal risk of progression to MN (180). Critical predictors of the 10-year probability of developing MN include high risk mutations, single *DNMT3A* mutations, presence of 2 or more mutations, age ≥65 years, diagnosis of CCUS or CHIP, maximum VAF ≥0.2, mean corpuscular volume (MCV) ≥100 fl, and red cell distribution width (RDW) ≥15% (180). For patients who are estimated to be at high risk of progression to MN, it is advisable to initiate regular monitoring of blood counts. If abnormalities are detected, the threshold for performing bone marrow biopsy should be low, and repeated deep sequencing should be considered for monitoring the clonal dynamics of CHIP. If a mutation has a VAF of ≥10%, or if there are ≥2 mutations present, it is recommended to conduct differential blood counts every 3 months in the first year and perhaps annually from the second year onward (181). If there is no evidence of progression, regular health check-ups are still advisable (181). Individuals with CHIP should also be evaluated for potential participation in a clinical trial aimed at preventing progression. Such trials are important to corroborate potential treatment options, such as administering high-dose vitamin C to restore gene function in *TET2-*mutated individuals (170). Since we do not yet have convincing therapies, clinical guidelines are also lacking.

Regarding cardiovascular risk management, the presence of a *JAK2^V617F^* mutation and events involving *JAK2-*mutated CN-LOH, for instance, are strongly associated with an increased risk of CVD (179). Somatic mutations in the three most frequently mutated CHIP genes, *DNMT3A, TET2* and *ASXL1*, have also been linked to a variety of cardiovascular problems. Certain CHIP mutations may be associated with lifestyle habits. For example, mutations in *ASXL1* seem to be promoted by smoking (38, 94, 96). Therefore, it should be helpful to counsel high-risk patients regarding lifestyle factors, in order to ameliorate the development of cardiovascular complications. Another approach is to suppress certain inflammatory pathways, in order to reduce the risk of CVD in patients with a high pro-inflammatory profile. Examples for that approach are the CANTOS (166, 167) and COLCOT (169) studies, using canakinumab and colchicine, respectively. In the future, such treatments may become available in the context of a CHIP clinic.

Counseling for CHIP becomes even more complex when germline mutations are incidentally detected along with somatic CHIP mutations. The potential impact on family members is considerable. According to ethical guidelines, it is ultimately the affected individual’s decision whether or not they prefer to be informed about such findings. They also retain the autonomy to decide whether or not their healthcare provider should convey the information to their relatives, who might carry the same germline mutation.

It has already been shown that interest in CHIP testing is high in certain patient groups. A study involving over 500 young breast cancer survivors gauged their perspective on testing for CHIP mutations. Being presented with a case vignette, 78.6% of respondents opted for CHIP testing (182). The interest in testing increased to 85.8% if the possibility of consultation in a specialized CHIP clinic was introduced, and further rose to 92.7% if the scenario included the availability of a hypothetical CHIP treatment (182). Patients expressed a strong desire to receive information about CHIP through printed material (62.7%) or website-based resources (60.2%) (182). Above all, in-person discussions, particularly with oncologists or cancer specialists, were the preferred mode of communication (88.1%) (182). Upon receiving a CHIP diagnosis, patients indicated their interest in periodic communications, lifestyle modifications, annual blood tests, and possible treatment (182). The preferred setting for CHIP follow-up care was predominantly a specialized breast oncology clinic (39.6%) or a CHIP-focused clinic (32.8%) (182). The levels of anxiety when learning about CHIP varied. Specifically, 26.9% reported no anxiety, 37.9% experienced mild anxiety, 24.4% had moderate anxiety, 2.5% indicated severe anxiety, and only 1.3% reported very severe anxiety (182). In summary, patients showed a keen interest in obtaining information about CHIP from specialized providers, along with receiving individualized risk assessment and health recommendations (182).

## Conclusion and outlook

CHIP is a fascinating field of research that expands our understanding of the development of hematologic disorders and cardiovascular risks. Several studies have demonstrated that CHIP can be a precursor to MDS and AML, in particular after cytotoxic therapy, suggesting that such treatments promote the emergence and expansion of mutated cells, thereby increasing the risk of leukemic transformation. However, only 0.5-1% of people affected by CHIP will develop a hematological malignancy. Interestingly, CHIP turned out to be associated with diverse cardiovascular diseases. Abnormal interaction between mutant monocytes-macrophages and the endothelium, along with increased expression of pro-inflammatory molecules like IL-1ß and IL-6, substantially promotes atherosclerotic development and also affects the myocardium, resulting in MI and/or HF. Since people with premature CAD do not exhibit traditional risk factors, they should be investigated for CHIP as a potential explanation for their cardiovascular problem. This requires deep sequencing of peripheral blood samples.

**Figure 1:**
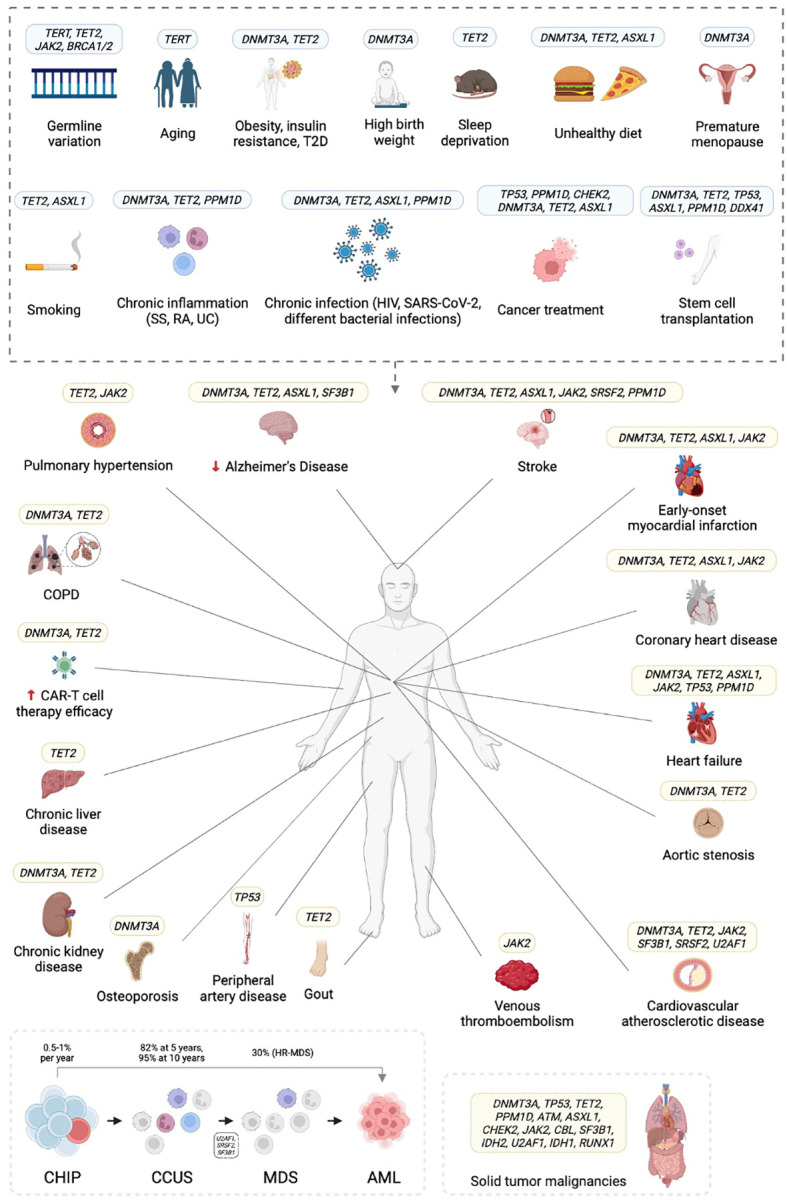
Clonal hematopoiesis of indeterminate potential (CHIP) is associated with several risk factors and confers hematologic, cardiovascular, and other risks. T2D, type 2 diabetes; COPD, chronic obstructive pulmonary disease; SS, systemic sclerosis; RA, rheumatoid arthritis; UC, ulcerative colitis; HIV, human immunodeficiency virus; SARS-CoV-2, severe acute respiratory syndrome coronavirus type 2; CAR-T, chimeric antigen receptor (CAR)-T cell therapy; CCUS, clonal cytopenia of undetermined significance; (HR-)MDS, (high risk) myelodysplastic syndrome; AML, acute myeloid leukemia. Created in BioRender.com.

A better understanding of the impact of specific mutations, their interactions with environmental factors, and their role in disease progression will help to identify individuals at high risk of hematological and/or cardiovascular complications and will foster the development of potential therapeutic and preventive strategies. Recurring patterns have already been identified in various CHIP-associated conditions (**Figure 1**).

Currently, treatment strategies for CHIP beyond the modification of traditional risk factors are limited. By targeting specific molecular pathways and inflammatory processes, it may be possible to mitigate the increased risk of cardiovascular events associated with the clonal expansion of abnormal myeloid cells. Besides neutralizing inflammatory mediators such as IL-1ß or IL-6, targeted inhibition of specific candidate driver mutations in CHIP, such as *TET2* or *JAK2*, might be pursued. However, further research is needed to validate these approaches.

Individuals with CHIP should receive proper counseling, which may best be provided in a specialized CHIP outpatient clinic. We and others are moving in that direction. Understanding the mutational landscape and associated clinical implications of CHIP variants is crucial for risk stratification and personalized management, taking into consideration individual lifestyle factors. We are going to focus our scientific interest on patients with early-onset cardiovascular diseases, hematological patients with suspected but not confirmed MDS, and breast cancer patients with *BRCA1/2* germline mutations who have received chemotherapy and/or PARP inhibitor therapy. Such patients will be screened for CHIP-related mutations, and those identified with CHIP will receive counseling in our CH outpatient clinic according to the current state of knowledge.

## Author contributions

AC: Writing – original draft, Writing – review & editing. FS: Writing – review & editing. UG: Supervision, Writing – review & editing. SD: Writing – review & editing. NG: Supervision, Writing – review & editing.
